# Cardioprotection of Controlled and Cardiac-Specific Over-Expression of A_2A_-Adenosine Receptor in the Pressure Overload

**DOI:** 10.1371/journal.pone.0039919

**Published:** 2012-07-06

**Authors:** Eman A. Hamad, Weizhong Zhu, Tung O. Chan, Valerie Myers, Erhe Gao, Xue Li, Jin Zhang, Jianliang Song, Xue-Qian Zhang, Joseph Y. Cheung, Walter Koch, Arthur M. Feldman

**Affiliations:** 1 Department of Physiology, Cardiovascular Research Center, Temple University School of Medicine, Philadelphia, Pennsylvania, United States of America; 2 Department of Medicine, The Center for Translational Medicine, Jefferson Medical College, Philadelphia, Pennsylvania, United States of America; Brigham and Women’s Hospital, United States of America

## Abstract

Adenosine binds to three G protein-coupled receptors (R) located on the cardiomyocyte (A_1_-R, A_2A_-R and A_3_-R) and provides cardiac protection during both ischemic and load-induced stress. While the role of adenosine receptor-subtypes has been well defined in the setting of ischemia-reperfusion, far less is known regarding their roles in protecting the heart during other forms of cardiac stress. Because of its ability to increase cardiac contractility and heart rate, we hypothesized that enhanced signaling through A_2A_-R would protect the heart during the stress of transverse aortic constriction (TAC). Using a cardiac-specific and inducible promoter, we selectively over-expressed A_2A_-R in FVB mice. Echocardiograms were obtained at baseline, 2, 4, 8, 12, 14 weeks and hearts were harvested at 14 weeks, when WT mice developed a significant decrease in cardiac function, an increase in end systolic and diastolic dimensions, a higher heart weight to body weight ratio (HW/BW), and marked fibrosis when compared with sham-operated WT. More importantly, these changes were significantly attenuated by over expression of the A_2A_-R. Furthermore, WT mice also demonstrated marked increases in the hypertrophic genes β-myosin heavy chain (β-MHC), and atrial natriuretic factor (ANF) – changes that are mediated by activation of the transcription factor GATA-4. Levels of the mRNAs encoding β-MHC, ANP, and GATA-4 were significantly lower in myocardium from A_2A_-R TG mice after TAC when compared with WT and sham-operated controls. In addition, three inflammatory factors genes encoding cysteine dioxygenase, complement component 3, and serine peptidase inhibitor, member 3N, were enhanced in WT TAC mice, but their expression was suppressed in A_2A_-R TG mice. A_2A_-R over-expression is protective against pressure-induced heart failure secondary to TAC. These cardioprotective effects are associated with attenuation of GATA-4 expression and inflammatory factors. The A_2A_-R may provide a novel new target for pharmacologic therapy in patients with cardiovascular disease.

## Introduction

Adenosine is an endogenous purine nucleoside that plays an important role in protecting the heart during ischemia. The cardiovascular effects of adenosine (A) are mediated by 4 G-protein–coupled receptors (A_1_-R, A_2A_-R, A_2B_-R and A_3_-R), all of which are expressed in the heart. Activation of A_2A_ -Rs results in coupling to G_s_ proteins and activation of adenylyl cyclase [1], [2], [3] while activation of the A_1_- and A_3_-Rs inhibits adenylyl cyclase and modulates other signaling pathways regulated by G_i/o_. Studies using murine models in which the A_1_- and A_3_-Rs have been genetically manipulated demonstrate a critical role for these receptors in cardiac protection during ischemia and reperfusion. [4], [5] By contrast, A_2A_-Rs have been shown to promote post ischemic protection through inhibition of inflammatory responses. [6], [7].

Owing at least in part to its pharmacological effects on neurohormone and cytokine activation, [8], [9] adenosine also affects ventricular remodeling in models of heart failure. For example, adenosine attenuates detrimental chamber remodeling in rodents with pressure overload hypertrophy and decreases cell size in cultured neonatal cardiomyocytes. [10], [11], [12], [13] However, the role of adenosine receptor-subtypes in cardiac remodeling has not been fully elucidated. Pharmacologic activation of the A_1_-R effectively attenuated the development of cardiac hypertrophy and prevented heart failure in mice that underwent transverse aortic constriction (TAC) [11] and mice that were A_1_-R gene-deficient had a higher mortality when compared with wild-type controls but did not demonstrate altered ventricular hypertrophy or increased cardiac dysfunction. [14] Surprisingly, mice in which the A_3_-R had been knocked out demonstrated an improved survival, decreased fibrosis and hypertrophy and a more robust left ventricular function after TAC when compared with wild-type controls. The role of the A_2_-R in cardiac remodeling has not been defined.

Previously, we demonstrated that constitutive and cardiac specific over-expression of the A_2A_ -R induced a hyper-contractile phenotype with enhanced calcium handling that prevented heart failure in a transgenic model [15]. This led us to hypothesize that signaling through the A_2A_ -R might also have salutary effects on cardiac remodeling. To test this hypothesis we assessed the effects of TAC on cardiac morphology, function and gene expression in wild type mice and in mice with cardiac specific and controlled (adult) over-expression of the A_2a_-R.

Sustained myocardial hypertrophy secondary to pressure overload is a leading cause in the development of heart failure and sudden death in humans [16], [17]. Hemodynamic overload is a complex physiological stimulus that can lead to marked changes in myocardial structure and function through various humeral and mechanical components. The hypertrophic response induced by pressure overload is associated with marked alterations in cardiac gene expression, which include reactivation of fetal gene expression patterns. Many studies demonstrated an increase in the expression of the fetal gene beta myosin heavy chain (β-MHC) as a sensitive marker for hypertrophy [18]. Many signaling pathways have been implicated in cardiac hypertrophy and subsequent failure. GATA-4 a cardiac restricted zinc finger transcription factor has been shown to control several genes up regulated during cardiac hypertrophy including β-MHC, cardiac troponin-C, atrial natriuretic factor, sodium/calcium exchanger (NCX), A_1_-R [19]. With that said, not all hypertrophy is thought to be deleterious. Animal models of hypertrophy have demonstrated adaptive hypertrophy with normalized wall stress and full compensation. For example, Insulin like growth factor (IGF) which has a signaling system involving Protein kinase B (PKB) has been described in an adaptive pressure induced process [20]. Athletes are thought to have physiologic hypertrophy secondary to endurance training, which is not associated with fibrosis or up regulation in hypertrophic response genes, and increases in wall thickness are modest.

## Results

**Figure 1:**
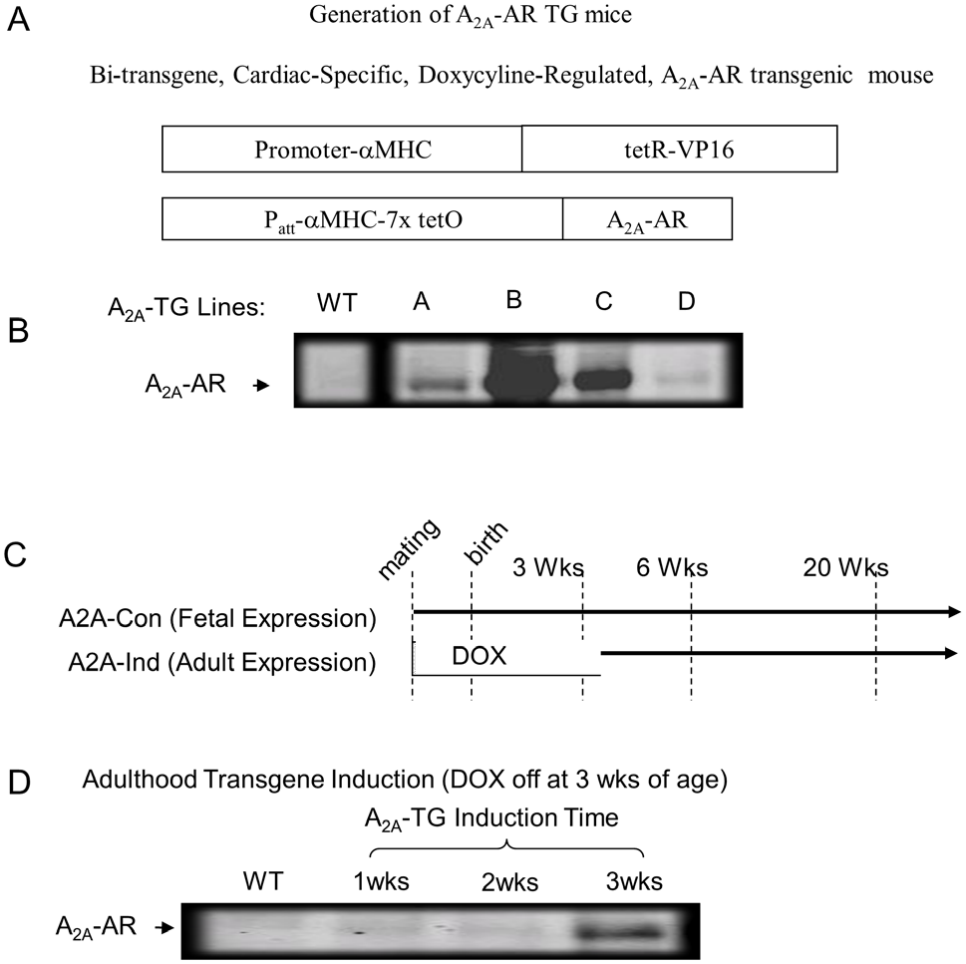
Over-expression of the A_2A_ adenosine receptor in mice myocardium. Mice with constitutive and controlled overexpression of A_2A_-R were created. (A & B) Bi-transgenic, cardiac specific doxycyline regulated A_2A_-R transgenic mice were generated and confirmed there is A_2A_-R expression in all of lines; (C & D) A representative diagram of the timeline of gene induction. The constitutive model was not placed on doxycyline and over expressed A_2A_-R at birth while the controlled or induced model was placed on doxcycline during mating and removed at the age of 3 weeks.

We created mice with inducible overexpression of A_2A_-AR. The human A_2A_-AR cDNA was cloned into a cardiac-specific and inducible controlled vector (TREMHC) composed of a modified mouse α-myosin heavy chain (α-MHC) minimal promoter fused with nucleotide binding sites for tetracycline transactivating factor (tTA) (**Fig. 1A**). [21] A_2A_-AR transgenic (TG) mice were engineered on an FVB background (PolyGene, Zurich, Switzerland) and crossed with mice that expressed tTA in the heart (MHC-tTA; **Fig. 1A**). In this “tetracycline-off” inducible system, the stable tetracycline analog doxycycline (DOX) inhibits tTA transactivation, and it was administered to mice at 300 mg/kg of mouse diet (Bio-Serv, Frenchtown, NJ). A_2A_-R transgenic founder lines expressing low and high levels of A_2A_-R as shown in Figure 1B, as evidenced by western blot. The constitutive model was not placed on doxycycline, while the induced model was placed on doxycycline during mating and removed after 3 weeks (**Fig. 1C**). As seen in Figure 1D, A_2A_-R was really detectable at 6-week-old mice by 3 weeks of induction. Mice generation was confirmed in our previous studies [22], [23], [24].

**Figure 2:**
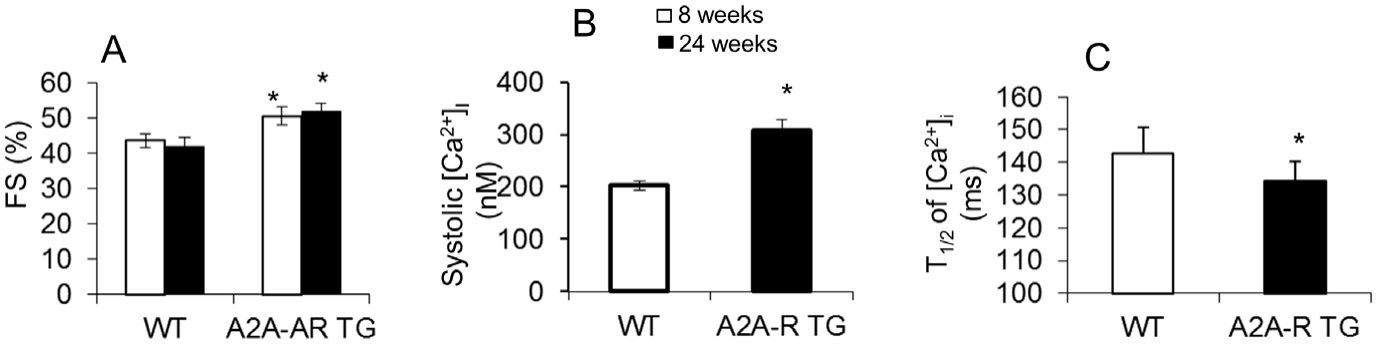
Effects of cardiac specific A_2A_-R e x**pression on cardiac funtion and calcium handeling.** (A) Echocardiography of mice with inducible, cardiac restricted expression of A_2A_-R TG and wild type (WT) mice. Fraction shorting (FS) at 8 week and 24 weeks in A_2A_-R TG and WT mice showed persist hyper-contractile phenotype in A_2A_-R TG mice up to 24 weeks (*p<0.01 vs WT mice, n = 8). (B & C) Calcium transient data showing increased systolic calcium (B) and rapid calcium re-uptake activity (C) in cardiomyocytes from the A_2A_-R TG mice at 10 weeks age compared to WT. Data were expressed as mean±SE. *p<0.01 compared to WT cardiomyocytes, n = 15 cells from 5 mice hearts.

At eight weeks of age, A_2A_-R TG mice demonstrated a significant increase in fractional shortening by 15–20% compared with non-transgenic littermates (Fig. 2A, P<0.05, n = 12), but were otherwise phenotypically normal. In contrast, heart rates and wall thickness were significantly increased in constitutive expression of A_2A_-R mice [23]. The increase in fractional shortening persisted at 24 weeks of age. The systolic intracellular Ca^2+^ in cardiac myoyctes from the mice at 10–12 weeks of age was significantly enhanced as seen in Fig. 2B (p<0.05, 15 cells from 5 mice hearts). At the same time, the recovery of intracellular Ca^2+^ were markedly rapid as shown in Fig. 2C (p<0.05, 23 cells from 5 mice hearts).

**Figure 3:**
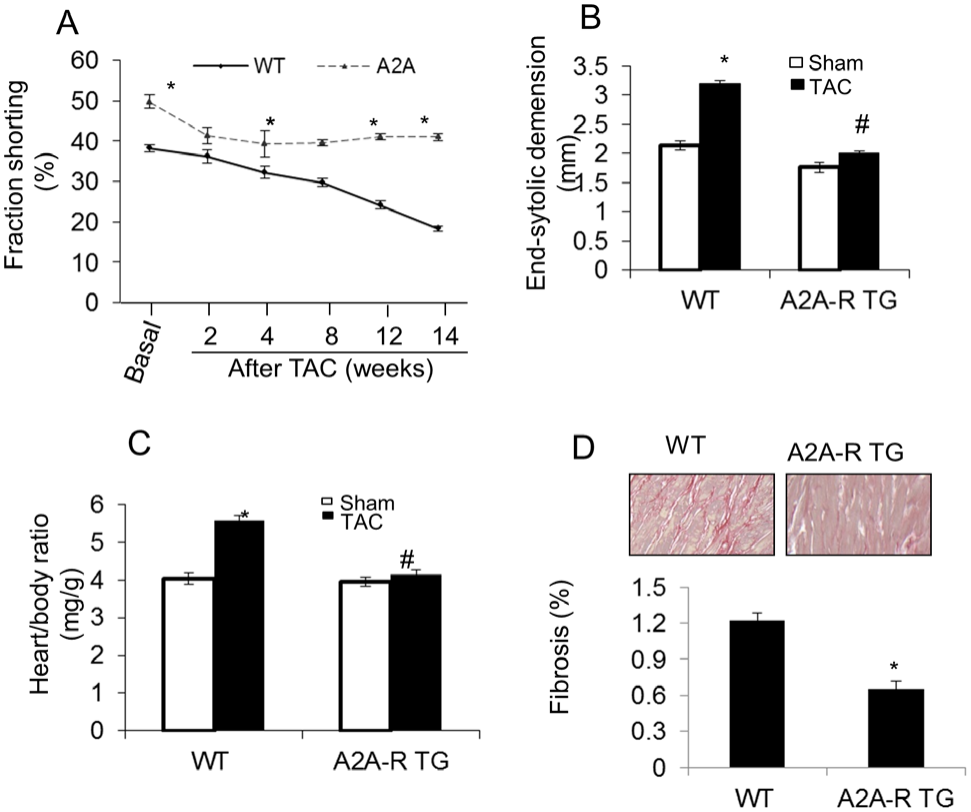
Effect of TAC on left ventricular hypertrophy and function as measured by Echocardiography. (A) A_2A_-R mice had preserved cardiac function and showed tolerance to TAC-induced pressure overload. WT mice developed a significant decrease in cardiac contractile function at 14 weeks after TAC (*P<0.01, repeated measures ANOVA test, n = 17). Of note, contractile function in A_2A_-R TG mice was slightly decline after TAC. *p<0.01 vs WT TAC at the same time point. (B &C) WT mice developed into a significant increase in end systolic dimensions (B) and a higher heart weight to body weight ratio (C).*p<0.01 vs sham group, n = 8–17; ^#^p<0.01 vs WT TAC, n = 10–17. (D) WT mice showed significantly more fibrosis than A_2A_-R TG at 14 weeks post TAC. Data was expressed as mean±SE *p<0.01 vs WT, n = 10–17. The basal fibrosis is no difference between WT and A_2A_-R TG. Both were below 0.1% of total myocardium area.

As expected, cardiac pressure overload by TAC caused a significant decrease in cardiac contractile function (Fig. 3A, Table 1) in WT mice. These changes could be seen as early as two weeks after TAC and persisted to the end experimental point at 14 weeks after TAC (p<0.001, n = 17, repeated measures two-way ANOVA test). The increase in end-systolic and end-diastolic dimension (Fig. 3B) and a higher heart weight to body weight ratio (HW/BW) (Fig. 3C) compared with sham-operated controls were attributed to the contractile dysfunction. More importantly, the development of left ventricular dysfunction (Fig. 3A & Table 2, p<0.01, n = 10–17), End systolic dimension (Fig. 3B & Table 2), heart/body ratio (Fig. 3C, p<0.01,n = 10–17), and cardiac fibrosis (Fig. 3D, p<0.01, n = 10–17) were markedly attenuated in mice with inducible, cardiac specific over-expressing A_2A_-R (Fig. 3A, 3B, 3C, 3D) mice at 14 weeks after TAC.

**Table 1:** Effect of TAC on LV hypertrophy and Function as Measured by Echocardiography.

	Baseline		2wks		4wks		8wks		14wks	
	WT	A_2A_ -R TG	WT	A_2A_ -R TG	WT	A_2A_ -R TG	WT	A_2A_ -R TG	WT	A_2A_ -R TG
***N***	17	10	17	10	17	10	17	10	17	10
**HR(b/min)**	470±11	538±11**	499±14	517±12	481±14	506±11	454±16	495±12	504±7	459±6
**FS %**	38.3±1.2	49.8±2.2**	36.2±1.2	41.9±1.2	32.2±2.0	39.3±3.2*	29.8±1.0	39.6±1.0**	18.4±0.7	40.9±0.9**
**LVEDD(mm)**	3.43±.09	3.40±0.08	3.47±0.09	3.2±0.09	3.54±0.09	3.43±0.08	3.63±0.06	3.29±.09*	3.92±.08	3.48±0.08**
**LVESD(mm)**	2.1±0.08	1.75±0.09*	2.23±0.01	1.79±0.09**	2.4±.09	2.2±0.15	2.56±.07	1.99±0.07**	3.2±0.05	2.02±0.04**
**AWT**	0.781±0.02	0.951±0.07	0.975±0.03	0.976±0.06	0.834±0.13	1.07±0.08	0.956±0.04	1.11±0.07	0.997±0.02	1.06±0.09
**PWT**	1.01±0.03	1.187±0.04	1.198±0.06	1.15±0.05	1.22±0.07	1.25±0.05	1.3±0.08	1.13±.06	1.084±0.04	1.17±0.08

HR: heart rate, beat/minute; FS: fraction shorting; LVEDD: Left Ventricular End Diastolic Diameter; LVESD: Left Ventricular End Systolic Diameter; AWT: anterior wall thickness; PWT: posterior wall thickness. Values are means ± SE. *p<0.05, **p<0.01 vs WT at the same time points. The data were assayed by repeated measures two-way ANOVA followed by Bonferroni multiple comparisons.

**Table 2:** Primers for Q-PCR.

Gene	Gene ID	Forward	Reverse
GATA-4	NM_008092	5′-CCA TCT CGC CTC CAG AGT-3′	5′-CTG GAA GAC ACC CCA ATC TC-3′
ANP	NM_008725	5′ CGT GCC CCG ACC CAC GCC AGC ATG G 3′	5′ GCC TCC GAG GGC CAG CGA GCA GAG C 3′
β-MHC	NM_080728	5′ - ACT GTC AAC ACT AAG AGG GTC A - 3′	5′ - TTG GAT GAT TTG ATC TTC CAG GG -3′
A1-R	NM_001039510	5′ AAC ATT GGG CCA CAG ACC TAC TTC 3′	5′ GAT GGA GCT CTG GGT GAG GAT GA 3′
β-actin	NM_007393	5′ GGA CCT GGC TGG CCG GGA CC 3′	5′ GCG GTG CAC GAT GGA GGG GC 3′
GAPDH	NM_008084	5′ AAC GAC CCC TTC ATT GAC 3′	5′ TCC ACG ACA TAC TCA GCA C 3′
Cdo1	NM_033037	5′-TCT GGT CTC TGA ACT CTA AT-3′	5′-TAG TCT CCA CAG CAT AGG-3′
C3	NM_009778	5′-CAT AGC CAA GTT CCT GTA-3′	5′-ATC TTC TTA TCG CCA TCC-3′
Serpina3n	NM_009252	5′-TGG TGC TGG TGA ATT ATA TC-3′	5′-GCG TAG AAC TCA GAC TTG-3′
Tlr7	NM_133211	5′-CTC TAC CTT GTG AAG TTA A-3′	5′-TAA GAT TGG TGG TGT TAG-3′

**Figure 4:**
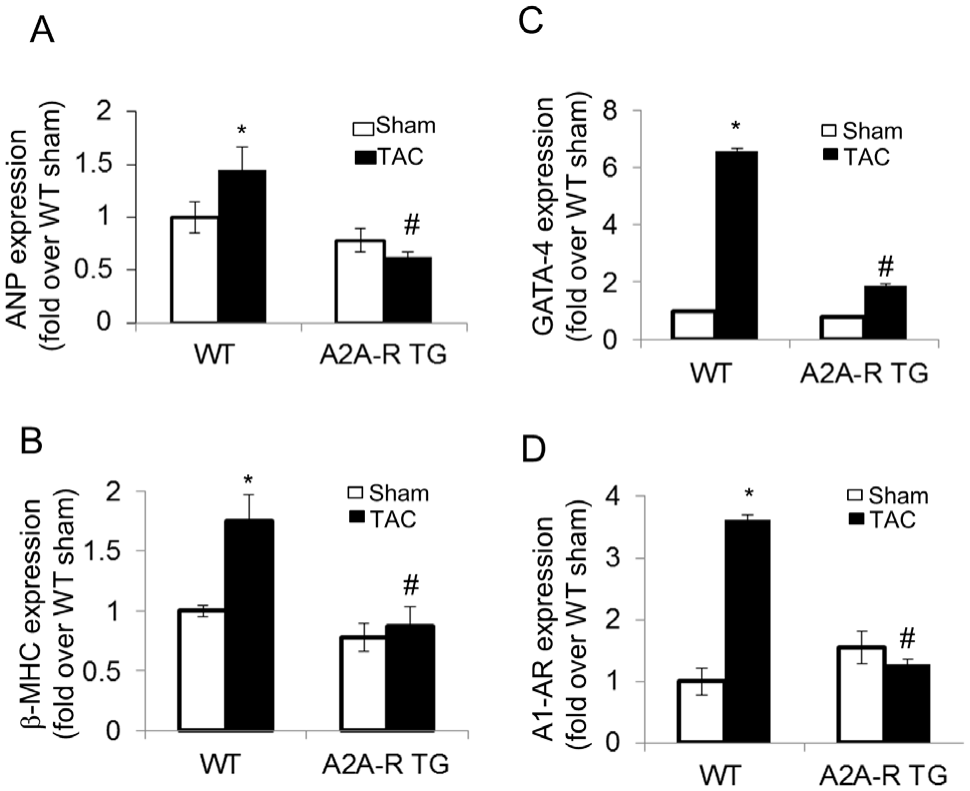
Effect of TAC on GATA-4, ANP, β-MHC and A1-R expression in A_2A_-R and WT mice. After 14 weeks of TAC, the total RNA was isolated from either A_2A_-R TG or WT mice myocardium. The Q-PCR was performed to check the gene expression of GATA-4 ANP, β-MHC and A_1_-R. Data were expressed as mean ± SE. ^∗^p<0.05 vs sham, ^#^p<0.05 vs WT mice TAC group, n = 6.

To assess the effects of pressure overload on gene expression in A_2A_-R TG and WT mice with or without pressure overload, we measured mRNA levels of the hypertrophic response genes ß-MHC and ANF as well as the transcription factor GATA-4. As seen in Figure 4, it was indeed that hypertrophic marker genes, the mRNAs encoding ANP (**Fig. 4A**, p<0.05, n = 7) and ß-MHC (**Fig. 4B**, p<0.05, n = 7), were significantly enhanced by 40.5±5.8% and 70.7±3.5%, respectively, in WT mice TAC group compared to sham group. Of note, these hypertrophic marker genes were dramatically suppressed in the inducible, cardiac-specific A_2A_-R TG mice (**Fig. 4A & 4B**). In addition, the mRNA encoding GATA-4, a transcription factor that mediates the activation of the hypertrophic gene program was expressed at a significantly lower level in A_2A_-R TG mice than that in wild type littermate controls after TAC (**Fig. 4C**, p<0.01, n = 7–8). Since overexpression of A_1_-R is known to cause a decrease in cardiac function [15], we measured the A_1_-R mRNA levels in both WT and A_2A_-R TG mice. As expected, the WT mice had a significant increase in A_1_-R levels 14 weeks (p<0.001 vs sham, n = 6) after TAC, but not in A_2A_-R TG mice (p<0.01 vs WT TAC group, n = 6), as shown in **Fig. 4D**.

**Figure 5:**
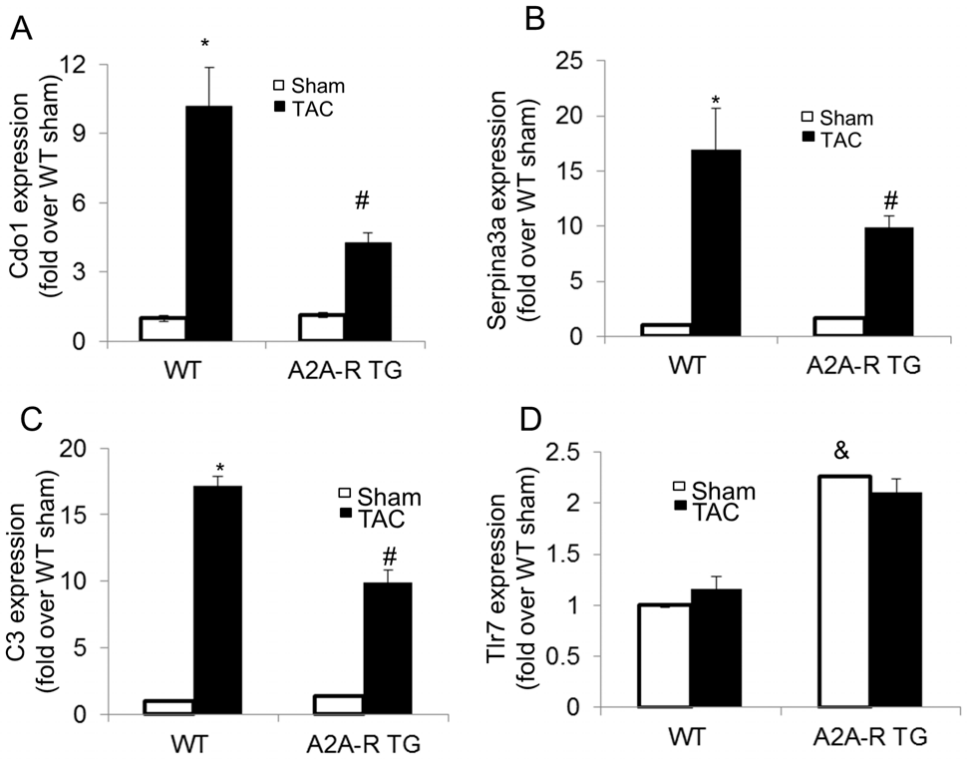
Effects of TAC on Inflammatory genes expression in A_2A_ and WT Mice. After 14wks of TAC, the total RNA was isolated from A_2A-_R TG and WT mice myocardium. The Q-PCR was performed to check the gene expression of cysteine dioxygenase 1 (Cdo1), complement component 3 (C3), serine (or cysteine) peptidase inhibitor, member 3N (Serpina3n), and toll-like receptor (TLr 7). Data was expressed as mean ± SE. *p<0.05 vs sham, ^#^p<0.05 vs WT mice TAC group, n = 6. ^&^p<0.05 vs WT sham.

Since it has recently been shown that cardiac inflammation are one of the major pathological factors involving in the pressure overload-induced murine heart failure [25], [26], [27] and activation of A_2A_-R are responsible for its anti-inflammatory effects [28], [29], we screened the experimental mice myocardium by gene microarray and validated the gene changes found in microarray by Q-PCR. As shown in Figure 5, cysteine dioxygenase 1 (Cdo1), complement component 3 (C3), and serine (or cysteine) peptidase inhibitor, member 3N (Serpina3n) were enhanced in WT TAC mice, but their expression were suppressed in A_2A_-R TG mice. Interestingly, toll-like receptor (TLr 7), which synergize with A_2A_-R agonists and adenosine to up-regulate VEGF, while simultaneously strongly down-regulating TNFα expression [30], was increased in A_2A_-R TG mice even without TAC (Fig. 5D).

## Discussion

The present study demonstrates for the first time that activation of the A_2A_-R signaling pathway can modulate the fibrosis, hypertrophy and subsequent left ventricular dysfunction that follow TAC using a murine model in which over-expression of the A_2A_ receptor can be controlled and is cardiac specific. This model system provides several unique features. Enhanced expression of the A_2A_-R: (1) is cardiac specific, thereby obviating effects of adenosine receptor signaling in the peripheral vasculature or in the central nervous system; (2) can be “controlled” in order to preclude the known effects of adenosine receptor signaling on cardiac and neural development; and (3) avoids the potentially confounding effects of using non-selective or partially selective adenosine receptor pharmacologic agonists and antagonists.

In concentric hypertrophy induced by pressure overload, it has been suggested that myocytes grow in width to increase wall thickness in order to regulate the pressure induced by increased wall stress [31], [32]. With sustained volume load, the compensatory hypertrophy transitions to heart failure and dilation. Many mechanisms have been implicated in this transition including, increased collagen and fibrosis, an upset in the balance between metalloproteinases and their inhibitors, oxidative stress and neurohormal activation [33]. In the present study the WT mice developed more fibrosis than the A_2A_-R TG mice after TAC.

These salutary affects of enhanced A_2A_-R signaling were associated with a marked attenuation in the expression of the hypertrophy-associated genes β-MYC and ANF and the transcription regulatory protein GATA-4. β-MHC is characterized by low adenosine triphosphate activity and low filament sliding velocity but can generate cross-bridge force with higher economy of energy consumption [34], [35], [36]. This suggests that up regulation of β-MHC can be an early adaptive response to pressure overload but over time leads to a decrease in contractile function [37]. Indeed, Dorn et al, suggested that depressed myocyte contractility after induction of pressure overload hypertrophy in aortic banded FVB mice is due in part to transcriptional up regulation of β-MHC [38]. GATA-4 has been shown to control several genes up-regulated during cardiac hypertrophy including β-MHC and ANF [19]. GATA-4 binding sites are thought to be required for activation of β-MHC and angiotensin II type a receptor expression - both of which have been implicated in pathological ventricular hypertrophy [39] and the over expression of GATA-4 generated cardiac hypertrophy in cultured cardiomyocytes and in mice. [40], [41] Thus the finding that the diminished hypertrophy and failure after TAC in the A_2A_-R TG mice is associated with a decrease in GATA-4 expression may imply a link between A_2A_-R signaling and the expression of hypertrophy genes. GATA-4 expression level varies between tissues, between developmental stages, and in disease states. Although it is often used as a marker of cardiomyocytes, in fetal heart GATA-4 expression is the highest in the proepicardium followed by endocardial cushions and then cardiomyocytes. GATA-4 expression in the adult heart had been reported to increase by approximately twofold in heart disease [42], [43]. However, little is known about the regulatory sequences that drive cardiac GATA-4 expression. Interestingly, Gs-protein coupled β-AR promotes GATA-4 signaling associated with cardiac hypertrophy [44], [45], [46], [47]. By contrast, we reported here that A_2A_-R, another Gs protein-coupled receptors that also signals through activation of adenylate cyclase, appears to diminshe GATA-4 expression. However, by contrast with the β_1_-AR, the A_2A_-R can also mediate activation of MAPKs and PKC [48] with subsequent induction of hypoxia-inducible factor 1 [49]. Thus, it might be a PKA-independent pathway that suppresses GATA-4 expression in myocytes after A_2A_-R signaling. However, further studies will be required to test this hypothesis.

Earlier studies have suggested a role for adenosine in cardioprotection during pressure-induced stress. For example, treatment with dipyridamole, an adenosine uptake blocker that increases myocardial adenosine levels, attenuated chamber remodeling in rats with pressure overload hypertrophy. [10] Similarly, the adenosine analogue 2-chloroadenosine lowered both heart to body weight ratios and improved left ventricular fractional shortening in mice exposed to TAC. [10] Consistent with these studies, diminished extra-cellular adenosine production as a result of a genetic deletion of CD73 exacerbated left ventricular hypertrophy and dysfunction after pressure overload. [13] In vitro, all of three adenosine receptors blunt the phenylephrine-induced rat neonatal cardiomyocytes hypertrophy [12]. However, the role of the A_1_- and A_3_-adenosine receptors in protecting the heart from the stress of pressure overload remains less clear. Using the selective A_1_-adeonsine agonist N^6^-cyclopentyladeonsine (CPA), Liao et al found that A_1_-R signaling attenuated TAC-induced changes in left ventricular fractional shortening and heart to body-weight ratios in C57B6 mice. However, when the A_1_-R was genetically deleted, TAC had identical effects on ventricular hypertrophy and dysfunction. [14] Furthermore, deletion of the A_3_-R attenuated TAC-induced left ventricular hypertrophy, fibrosis and dysfunction, suggesting that over-expression of the A_3_-R would have a deleterious effect. Since A_1_- and A_3_-R signaling inhibit adenylyl cyclase, slow heart rate, and inhibit cardiac contractility while A_2A_-R signaling increases adenylyl cyclase activity and enhances cardiac contractility, it is not surprising that these different adenosine receptor-subtypes have disparate effects in the context of pressure-induced stress. [15].

A_2A_-R agonist displays rapid anti-inflammatory properties in a variety of in vitro and in vivo models [50], [51], [52]. And cardiac inflammation is one of the major pathological factors involving in the pressure overload-induced murine heart failure [25], [26], [27]. In the present study, four inflammatory factors are suppressed by over-expression of A_2A_-R, which might be attributable to its salutary effects on cardiac remodeling. Future studies will be required to determine why the enhanced cardiac specific A_2A_-R signaling suppresses myocardial inflammation and what the molecular relationship is between mycoytes, inflammatory cells, and fibroblast during enhanced A_2A_-R signaling.

In summary our study demonstrates that A_2A_-R over-expression is protective against pressure-induced heart failure secondary to TAC. These cardioprotective effects are associated with inhibition of GATA-4 expression and attenuation of the up-regulation of hypertrophy gene program that characterizes the pressure overloaded heart. Taken together, these results suggest that the A_2A_-R may be a therapeutic target in the treatment of patients with hypertension or hypertrophic heart disease.

## Materials and Methods

### Transgenic Mouse Generation

Experiments were carried out in transgenic mice with controlled cardiac restricted over expression of the human A_2A_-R TG as previously described [15]. Using a cardiac-specific and inducible promoter, we selectively over-expressed A_2A_-R TG in FVB mice after removal of doxycycline (DOX) from their diet at 3wks. Animal studies were approved by the Institutional Animal Care and Use Committee of Thomas Jefferson University.

### [Ca^2+^]_i_ Transient Measurements

Myocytes from A_2A_-R TG and WT mice were exposed to 0.67 µM of fura-2 AM for 15 minutes at 37°C. Fura-2-loaded myocytes were field-stimulated to contract (1 Hz, 37°C) in medium 199 containing 1.8 mM [Ca^2+^]_o_. Fura-2-loaded myocytes mounted on [Ca^2+^]_i_ transient measurements using a Dvorak-Stotler chamber situated in a temperature-controlled stage (37°C) of a Zeiss IM 35 inverted microscope (Thornwood, NY) were performed as previously described [53].

### Surgical Procedure for Transverse Aortic Banding

Eight-week-old male wild-type FVB mice (N = 17) and A_2A_ -R littermates (N = 10) underwent transverse aortic banding (TAC) as previously described [15]. Briefly, an aortic band was created by placing a ligature (7-0 nylon suture) securely between the origin of the right innominate and left common carotid arteries with a 27-gauge needle as a guide. The sham procedure was identical except that the aorta was not ligated. Each strain has 8 mice in Sham group.

### In Vivo Assessment of Cardiac Function

Left ventricular (LV) function was evaluated with transthoracic echocardiography at baseline, 2, 4, 8, 12, and 14Wks. A Visual Sonic Vevo 770 imaging system was used (Miami, FL). Mice were lightly sedated with isoflurane. A parasternal short-axis view was obtained for LV M-mode imaging at the papillary muscle level. Three independent M-mode images were used for measurements of LV end-diastolic internal diameter (LVEDD) and LV end-systolic internal diameter (LVESD) in two consecutive beats according to the American Society of Echocardiography leading edge method. Fractional shortening (FS) was calculated as FS%  = [(LVEDD – LVESD)/LVEDD]×100. Anterior (AWT) and Posterior Wall thickness (PWT) were also measured. Hearts were harvested at 14 weeks.

### Real-Time Polymerase Chain Reaction

Reverse-transcribed cDNA from myocardial mRNA was used to determine the expression of A_2_-AR, atrial natriuretic peptide (ANP), GATA-4, and β-MHC. cDNA was reverse transcribed from 1µg of total RNA extracted from the left ventricular myocardium of male mice (n = 6 for each group) with the primers as shown in table 2. GAPDH and actin genes were used as a reference for normalization of obtained measurements. Briefly, 40 ng of genomic DNA from mouse tail was used to quantify the number of transgenes inserted into the genome. Analysis of gene expression was performed using 2(-delta delta C(T)) method. [54].

### Immunoblotting and Histopathology of Myocardium

Picrosirius red staining for assessment of fibrosis was performed by the Research Animal Diagnostic Laboratory (University of Missouri). To determine fibrosis, 5 independent high-power fields of stained images from each animal were analyzed by a blinded observer with Image-Pro Plus software (MediaCybernetics, Silver Spring, MD).

### Statistics

All results are expressed as means ± SE. Two-way analysis of variance was used to analyze the calcium transient. Repeated measurement ANOVA was used to analyze the contractile function after TAC. Commercial software package were used for all statistical analysis (Graph Pad, La Jolla, CA) two group comparisons were made with the unpaired student t-test. In all analyses, *p*<0.05 was taken to be statistically significant.
